# Facile Titrimetric Assay of Lysophosphatidic Acid in Human Serum and Plasma for Ovarian Cancer Detection

**DOI:** 10.15430/JCP.2023.28.2.31

**Published:** 2023-06-30

**Authors:** Nazia Tarannum, Deepak Kumar, Ranu Agrawal

**Affiliations:** 1 Department of Chemistry, Chaudhary Charan Singh University, Meerut, India; 2 Department of Applied Science, Sir Chhotu Ram Institute of Engineering and Technology, Chaudhary Charan Singh University, Meerut, India

**Keywords:** Acid-base, Titration, Lysophosphatidic acid, Concentration, Assay

## Abstract

Herein, an instrument free facile acid-base titrimetric methodology is reported for lysophosphatidic acid (LPA) measurement in serum and plasma samples for ovarian cancer detection. The concept is based on the titrimetric method in which alkaline solution was titrated with free fatty acid. Free fatty acid is generated due to action of the lysophospholipase to LPA. A phospholipid derivative known as LPA can function as a signaling molecule. A glycerol backbone serves as the foundation for phosphatidic acid, which also has bonds to an unsaturated fatty acid at carbon-1, a hydroxyl group at carbon-2, and a phosphate molecule at carbon-3. Free fatty acid and glycerol-3-phosphate are formed when LPA reacts with lysophospholipase. The formation of free fatty acid depends on the concentration of LPA. The standard graph of known concentrations of LPA, LPA spiked serum and LPA spiked plasma was plotted. The concentration of LPA in unknown serum and plasma were calculated from the standard graph. The limit of detection of LPA in spiked serum and plasma samples via titrimetric assay was calculated as 0.156 μmol/L. A patient's chance of survival may be outweighed by an early diagnosis of ovarian cancer.

## INTRODUCTION

**Figure 1:**
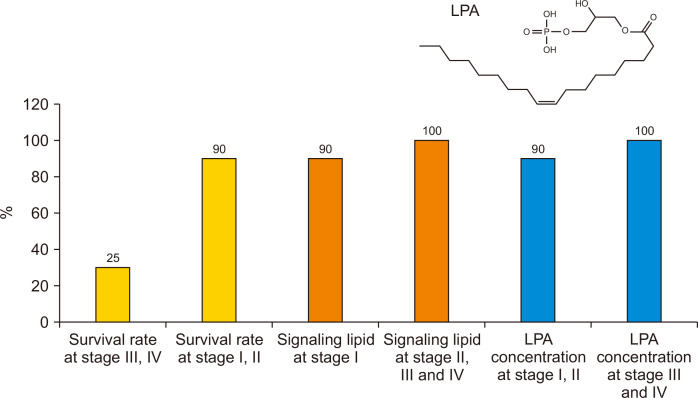
Survival rate, signaling lipid, and lysophosphatidic acid (LPA) concentrations at different stages of ovarian cancer.

Cancer kills more than 15% of women over 50, making it one of the main causes of death [1,2]. Ovarian cancer is one of the most serious tumors that affect women. While ovarian cancer is less frequent than several other malignancies of females, like breast cancer, it has the most related death ratio of all gynecological cancers, particularly for post-menopausal women [3-7]. When compared to women diagnosed in stages I or II of the disease who have a survival rate of five-year of over 90%, late-diagnosed women have a poor five-year survival rate of only 25% [8-10]. This information makes it abundantly evident that, in order to greatly improve the survival rate for those women who have the condition, improved diagnosis in early stages of a disease is fundamentally necessary. Lysophosphatidic acid (LPA), as shown in Figure 1, is a signaling lipid mediator [11], and is a potential biomarker for ovarian cancer. The ovarian cancer activating factor comprises various species of LPA. LPA stimulates the proliferation of cancer cells, intracellular calcium release, and tyrosine phosphorylation. This suggests that ovarian cancer activating factor or LPA may play a prominent role in ovarian cancer cell growth.

LPA is a highly specific marker for early-stage ovarian cancer and has great prospects for clinical applications [12]. The LPA has been shown to be a multifunctional signaling molecule in fibroblasts and other cells. In two different studies, it was discovered that this signaling lipid was raised 90% in stage I ovarian cancer patients and 100% in individuals with the later-stage disease [13,14]. Additionally, it was discovered that the elevated serum LPA concentration is linked to the disease stage, with III and IV stage patients having a greater value than the stage I and II patients, as seen in Figure 1. Based on these observations, it is considered that LPA is an extremely promising marker for the early-stage identification of ovarian cancer.

LPA is measured using conventional analytical techniques. These techniques include capillary electrophoresis, UV detection [15,16], matrix-assisted laser desorption/ionization mass, gas chromatography combined with mass spectrometry [13,17-19], liquid chromatography with mass spectrometry [14,20-24] or absorbance spectroscopy [25], thin-layer chromatography combined with mass spectrometry [26-30] and ELISA assay. However, since they require specialized instruments and a difficult technique, like extraction of lipid and LPA isolation from the other lipids in serum, the majority of these LPA assay procedures are not regularly utilized for diagnosis. For routine LPA analysis, it is required to develop a sustainable and low-cost approach.

Due to their inadequacy for practical clinical analysis and the need for new techniques with simpler procedures, there is a need for a development of a titrimetric method for ovarian cancer diagnosis. The present study describes the titrimetric process for the determination of free fatty acids formed by hydrolysis of LPA in serum and plasma samples. Free fatty acids are formed when LPA reacts with lysophospholipase enzyme. This concentration of free fatty acids depends upon the concentration of LPA. It is a facile, economic, and no instrument requiring approach for ovarian cancer diagnosis.

Titration of acid or base is a technique for evaluating the unknown concentration of a solution of acids or bases by neutralizing them with the known concentration’s solution of bases or acids, respectively. In this method, an indicator is used that changes the colour at the end of titration. This method allows the quantitative approach for the measurement of unknown acid/base concentrations. Van der Schoot et al. [31] and Guenat et al. [32] suggested use of the sensor actuator using electrochemical reduction of water on the surface of the electrode to produce protons, which led to the following acidification of the water. In more recent work, Steininger et al. [33] showed how to determine the buffer capacity of many samples by swapping the electrochemical pH detection.

Many carbon materials, including MWNTs [34-36], have their acidic and basic characteristics determined using acid-base titration methods [37-40]. For the purpose of measuring the concentration of free fatty acid in biological matrices, several studies have been performed [41-45]. Mohammed et al. [46] has described an acid-base titrimetric method for sildenafil citrate determination in a tablet and bulk form. More recently, Wiorek et al. [47] reported reagentless electroanalytical technology for automated titrations of acid-base of water samples.

In the present work, the concentration of LPA in plasma and serum samples was measured by the acid-base titrimetric method based on the formation of free fatty acids. In this method, standard solution of sodium hydroxide (NaOH), (alkaline) was prepared, and phenolphthalein was used as an indicator. The technique relies on the observation of the indicator’s pink color disappearance due to the neutralization of NaOH, which was employed as a carrier stream even by inserted free fatty acid sample. The standard graph of known concentrations of LPA, LPA spiked serum and LPA spiked plasma was plotted. The concentrations of LPA in unknown serum and plasma were calculated from the standard plot.

## MATERIALS AND METHODS

### Experimental design

#### 1) Chemicals and reagent

PBS buffer (pH 7.4), oxalic acid, phenolphthalein and NaOH were purchased from Central Drug House. LPA, Lysophospholipase (EC 3.1.1.5) and Triton X-100 were purchased from Sigma Aldrich.

#### 2) Collection of blood samples

Blood samples were collected from different age groups ranging from 21 to 60 years. Around 50 samples were collected from different aged healthy female volunteers. The blood samples were divided into 5 age groups as shown in Table S1. In Table S2, the three different blood samples were analyzed in females of 21 to 30 years of age. Blood analysis is one of the most important tools available to clinical healthcare. Its data rely upon the clinical setting for interpretation of clinical signs and symptoms.

#### 3) Preparation of working solutions

The solution 0.01 N NaOH was prepared in water. The solution of oxalic acid (0.01 N) was prepared in water. The indicator (phenolphthalein) was prepared in ethanol.

#### 4) Preparation of serial dilutions of LPA in PBS, serum, and plasma

LPA standard stock solution (20 µmol) was formed by dissolving 9 mg LPA in 10 mL PBS buffer solution. Then LPA stock solution was serially diluted to 0.156, 0.312, 0.625, 2.5, 5, 10, and 20 µmol/L in PBS buffer (pH 7.4).

The blood samples were collected, and plasma and serum were isolated from the blood. LPA stock solution (20 mol) was used to spike the serum and plasma sample and diluted to final standard concentrations viz., 0.156, 0.312, 0.625, 2.5, 5, 10, and 20 µmol/L. Before titration, each LPA sample was first incubated with lysophospholipase for 15 minutes to obtain free fatty acids.

#### 5) Procedure of titrimetric method

**Figure 2:**
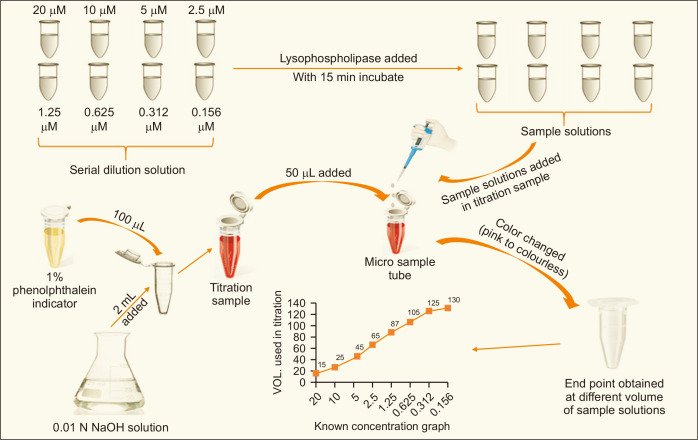
Titrimetric method of acid-base with serial dilution solution and sodium hydroxide (NaOH) standard solution.

The actual concentration of 0.01N NaOH was calculated using primary standard oxalic acid solution titrated by NaOH solution using phenolphthalein as an indicator. An accurate volume 50 µL of 0.01 N NaOH standard (2 mL) was taken in a sample tube, and 100 µL of 1% phenolphthalein indicator was added to it and titration was carried out against serial dilution solutions of LPA in different medium viz., PBS, serum, and plasma. The LPA was added to the sample tube containing NaOH and phenolphthalein with the help of micropipette (10 µL). Reading was taken at the end point where pink colour changed to colourless. The readings were recorded for each LPA sample solution. Figure 2 illustrates the acid-base titration procedure with different LPA concentrations.

#### 6) Analysis of unknown serum and plasma samples

**Figure 3:**
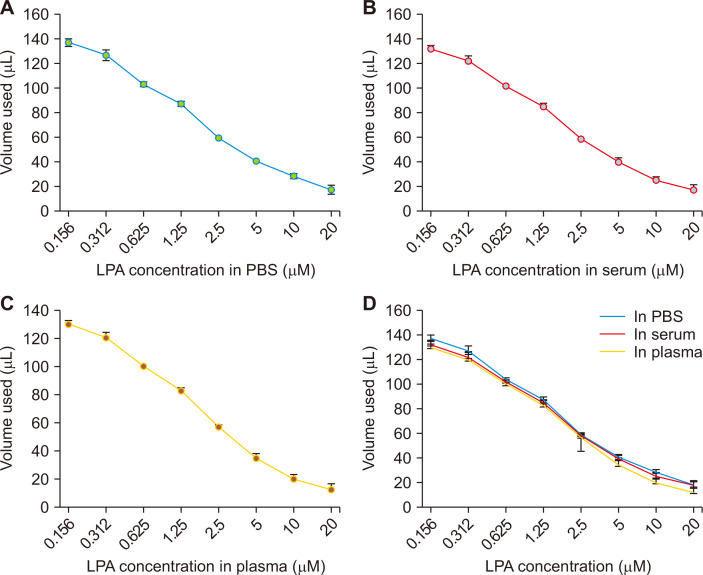
Standard graph plot of known lysophosphatidic acid (LPA) concentrations in serial dilutions of (A) PBS, (B) spiked serum, (C) spiked plasma and (D) comparison between the LPA concentration in PBS spiked serum and spiked plasma with volume used in titration.

Blood samples were taken from healthy female volunteers (age between 21 to 60 years) subject to analysis for LPA concentrations. The serum and plasma were isolated from given blood samples. The standard graph plotted with spiked LPA in serum and plasma (Fig. 3) was used to extrapolate the concentration of LPA in serum and plasma samples, respectively. The blood samples of same age healthy female volunteers were also compared for LPA concentrations.

#### 7) Statistical analysis

All data were expressed in the form of mean ± standard deviation and analysed by one way ANOVA for the significance at *P*-value 0.05. Standard deviation analysed by one way ANOVA in MS excel software office (Microsoft).

## RESULTS

### Chemistry of the titrimetric assay

**Figure 4:**
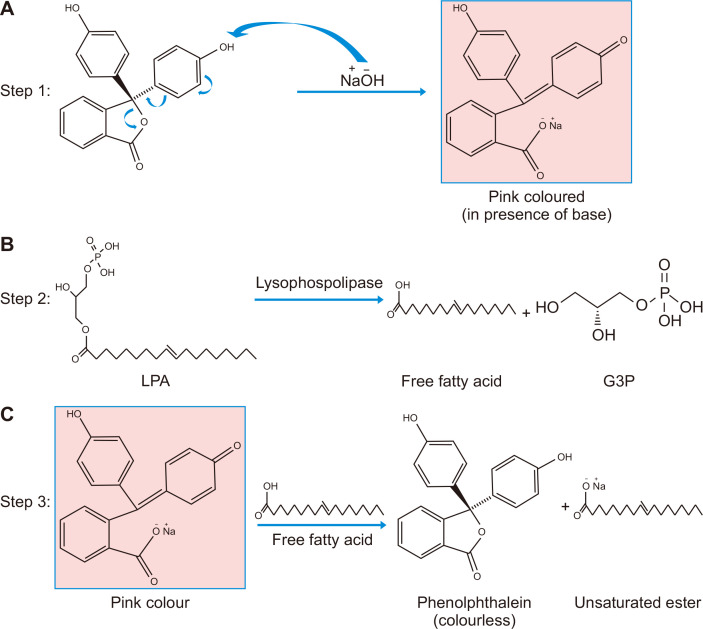
Chemistry of titrimetric assay. (A) Step 1 shows change in colour of indicator. (B) Step 2 shows formation of free fatty acid after reaction of LPA with lysophospholipase. (C) Step 3 shows titration of free fatty acid with base in the presence of the indicator. LPA, lysophosphatidic acid; NaOH, sodium hydroxide.

NaOH acts as a base and reacts with the OH group of phenolphthalein. NaOH withdraws a proton from the OH group of phenolphthalein as the OH group delocalizes the benzene ring and five membered lactone ring opens with cocomitant changes of colourless phenolphthalein to pink colour (as shown in Fig. 4 step 1). In another step, LPA is incubated for 15 minutes with lysophospholipase to form free fatty acid and glycerol-3-phosphate as shown in Figure 4 step 2. Now, phenolphthalein (pink colour) is titrated with free fatty acid in which the ester group of phenolphthalein reacts internally with the quinone ring in the presence of free fatty acid. The Na+ ion become combined with the ester bond of free fatty acid and the sodium salt of free fatty acid is formed. The pink colour of phenolphthalein changes to colourless at the neutralization point.

### Standard graph of the LPA concentration

The graph was plotted with LPA concentrations (µM) at X-axis and volume of LPA (treated with lysophospholipase) used for neutralization of NaOH was taken on Y-axis. These standard graphs were plotted with LPA concentrations in spiked PBS, serum, and plasma as shown in Figure 3. The limit of detection of LPA in spiked plasma and serum samples was calculated as 0.156 µmol/L.

### Standardization of analytical parameters in titration

LPA is treated with lysophospholipase for certain incubation time to release free fatty acids. Since in this titrimetric analysis, the amount of base used is dependent upon the concentration of free fatty acids, the amount of the lysophospholipase enzyme and reaction incubation time were optimized to obtain maximum free fatty acids.

### Standardization of incubation time

**Figure 5:**
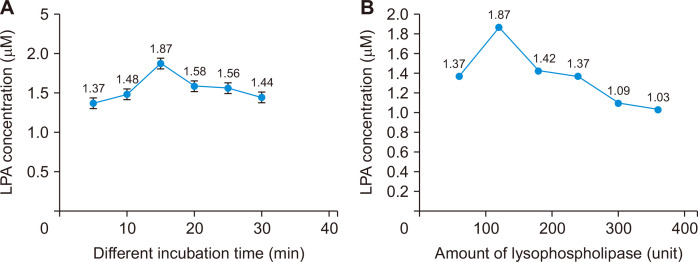
Optimization of the lysophosphatidic acid (LPA) concentration with (A) different incubation times and (B) different amounts of lysophospholipase.

Lysophospholipase (120 unit) was added to serum for different incubation times i.e., 30, 25, 20, 15, 10, and 5 minutes, and titration was performed for the determination of LPA concentrations. As shown in Figure 5A, the development of free fatty acids was increased with the increase in incubation time, and the endpoint was achieved in 15 minutes of incubation.

### Standardization of the lysophospholipase concentration

The amount of lysophospholipase used to release free fatty acid was also optimized. In this experiment, the reaction was performed in the presence of varying amounts of lysophospholipase (60 unit, 120 unit, 180 unit, 240 unit, 300 unit, and 360 unit). The result is shown in Figure 5B; maximum quantity of free fatty acid was released in the presence of 120 unit of lysophospholipase.

### Effects of thawing and freezing of serum/plasma samples on the concentration of LPA

The thawing and freezing cycles of serum and plasma samples may affect the concentration of LPA. The experiments were conducted to determine the negative impacts of freezing and thawing on the LPA concentration. The LPA concentration was measured following 1 to 5 cycles of thawing and freezing.

### Titrimetric assay of unknown serum and plasma samples

**Figure 6:**
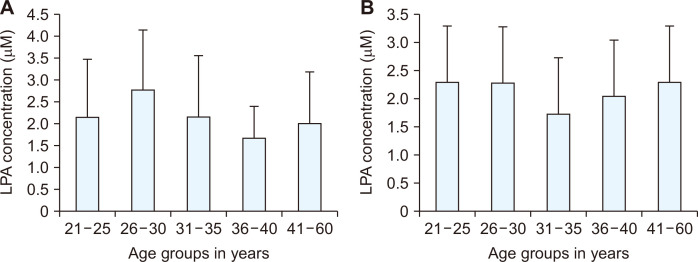
Lysophosphatidic acid (LPA) concentrations in (A) serum sample and (B) plasma sample of different age groups.

Blood samples were taken from 25 healthy female volunteers (age between 21 to 60 years). These samples were categorized into 5 groups (Fig. 6) to measure the LPA concentration through titrimetric analysis. The serum and plasma were isolated from a blood sample. The standard graph plotted with spiked LPA in serum and plasma (Fig. 3) was used to dictate the LPA concentration in unknown plasma and serum samples shown in Table S1 and S3. Figure 6 shows concentrations of LPA in unknown plasma and serum samples measured by the titrimetric method.

### Quantitative analysis of the concentration of LPA in serum samples of volunteers of the same age

**Figure 7:**
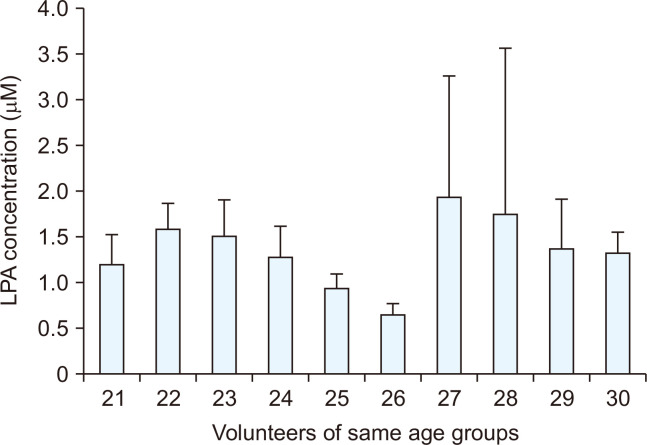
Lysophosphatidic acid (LPA) concentrations in serum samples of three volunteers of same age groups (21-30 years).

The 30 blood samples were collected from healthy female volunteers (21-30 years) and serum was isolated. Three samples were collected from each age group and analyzed. The titrimetric method as discussed above was used to measure LPA concentrations in serum samples of the same age group volunteers shown in Table S2. The LPA concentrations in serum samples were measured by extrapolation on standard graph (Fig. 3) shown in Figure 7.

### Comparison of concentrations of LPA in serum and plasma samples of same age female volunteers

Plasma and serum were isolated from the same blood sample of healthy female volunteers (Fig. 7). As LPA is found in both serum and plasma, the comparative study was done to measure LPA concentrations in serum and plasma samples as well by the titrimetric method. The serum and plasma were isolated from 10 blood samples taken from healthy female volunteers of different age groups (21-30 years) and analyzed for the quantitative determination of LPA.

## DISCUSSION

The acid-base titrimetric assay is facile, economic, and instrument less, and requires relatively little time. This assay confirms the critical level of LPA in serum and plasma samples which may help to diagnose at an early stage of ovarian cancer. The standard graph of LPA concentrations was plotted with the LPA concentrations (µM) at X-axis and the volume of LPA (treated with lysophospholipase) used for neutralization of NaOH on Y-axis. The data revealed that the equal volumes of LPA spiked serum and LPA spiked plasma were used to obtain end point in the titrimetric method to neutralize free fatty acids. In this experiment, more amount of LPA spiked serum was used for the neutralization in comparison to the amount of LPA spiked plasma as illustrated in Figure 3. It may be due to the presence of more LPA in the plasma (non-spiked) in comparison to the serum (non-spiked). As soon as the concentration of LPA (after enzymatic hydrolysis converts in free fatty acids) in serum and plasma increased, the volume of acid used for neutralization decreased in the titrimetric method.

The amount of base used is dependent upon the concentration of free fatty acids, and the amount of lysophospholipase and reaction incubation time were optimized to obtain maximum free fatty acids. Standardization of incubation time shows that in Figure 5A. Lysophospholipase (120 unit) was added to serum for different incubation times (i.e., 30, 25, 20, 15, 10, and 5 minutes), and titration was performed for the determination of LPA concentrations. The generation of free fatty acids was increased with the increase in incubation time, and the endpoint was achieved in 15 minutes of incubation. Therefore, 15 minutes were considered as an optimum incubation period for the lysophospholipase reaction in further experiments.

**Figure 8:**
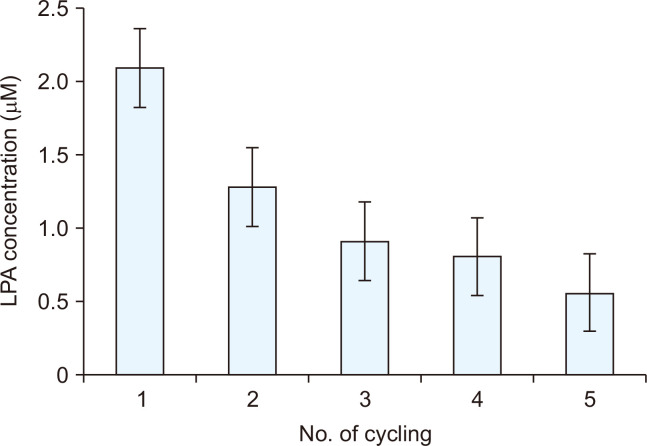
Effect of thawing and freezing cycles on the concentration of lysophosphatidic acid (LPA) in serum/plasma sample.

Now, the amount of the lysophospholipase enzyme used to release free fatty acid was also optimized. The data revealed that the concentration of free fatty acid formed increased with an increment in the amount of lysophospholipase added to samples. The maximum quantity of free fatty acid was created in the presence of 120 unit of lysophospholipase. On increasing the amount of lysophospholipase, the LPA concentration was found to decrease as shown in Figure 5B. This is probably due to the steric hindrance of more quantity of free fatty acids (product) formed. Hence, 120-unit lysophospholipase was considered an optimum concentration for the reaction and further experiments were done with this optimum value of lysophospholipase. Thawing and freezing effect of serum/plasma samples on the concentration of LPA was found to decrease after every cycle of freezing and thawing (Fig. 8). The data suggested that the LPA concentration decreased due to the degradation during the freezing and thawing cycle of serum and plasma samples.

**Figure 9:**
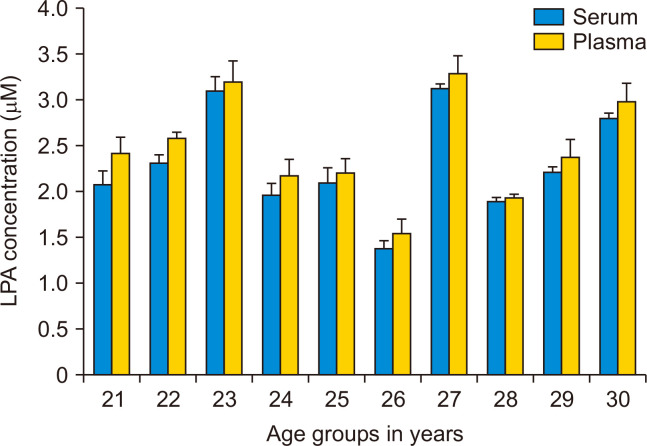
Lysophosphatidic acid (LPA) concentrations in serum and plasma of same age group volunteers.

Quantitative analysis of concentrations of LPA in serum samples of volunteers of the same age indicated that three volunteers of the same age had varying LPA concentrations, which were not noticeably different at the level of *P* ≤ 0.05 and showed the same LPA concentration. Further, there is no significant difference in the LPA concentration among different age groups, as shown in Figure 7. The serum and plasma were isolated from 10 blood samples taken from healthy female volunteers of different age groups (21-30 years) and subjected to the quantitative determination of LPA. Herein, the data shows that the levels of LPA in serum and plasma samples were noticeably different excepting the 23-, 25- and 28-years age group. The value of LPA concentrations in both samples is shown in Table S4. These results revealed that volunteers of the same age group as shown in Figure 9 have more LPA concentrations in plasma than serum samples excepting the 23-, 25- and 28-years age groups. The literature supports present study using several transducers, as shown in Table 1, to detect LPA in patients of ovarian cancer. The used transducers, sample source, and limit of detection are all listed in the Table 1 [48-53].

In this study, instrument free facile acid-base titrimetric methodology for LPA detection in serum and plasma samples for ovarian cancer detection was introduced. The method was based on titration of free fatty acid against 0.01M NaOH using phenolphthalein as an indicator. The concentration of LPA in plasma and serum samples was measured by the concentration of formed free fatty acid using the acid-base titrimetric method. The method was validated with respect to the linearity, precision, and accuracy. The second most frequent type of gynecological cancer is ovarian cancer, which is also the most prevalent type of gynecological cancer to cause death. The majority of women have advanced disease when they are diagnosed, which results in a poor prognosis even with effective therapy. In severe disease stages, a cure is uncertain. Even though chemotherapy has a high first response rate, new therapy options are necessary because most women experience recurring illness. The acid-base titrimetric assay is a facile, economic, instrument less method and requires little time. This assay confirms the critical level of LPA in serum and plasma samples which may help to diagnose at an early stage of ovarian cancer.

## Figures and Tables

**Table 1:** Various transducers, sample sources, and LOD employed to detect LPA in patients of ovarian cancer

Serial no.	Sample source	Linear/limit range/adsorption efficiency	Transducer used	Year	Reference
1	Serum	0.1-16 µM	HPLC-MS/NS method	2007	[48]
2	Serum/plasma	1.86 µM-11.53 µM	UV-vis spectroscopy	2011	[49]
3	Serum	-	Meta-analysis approach	2015	[50]
4	Plasma	0.14-1.64 µM	ELISA	2017	[51]
5	Plasma	0.5 µM	Synergistic tailoring	2017	[52]
6	Serum	5 µM	Fluorescence microscopy	2020	[53]
7	Serum/plasma	0.156 µM	Facile titrimetric assay	Current	Current

HPLC-MS, high performance liquid chromatography-mass spectrometry; LPA, lysophosphatidic acid; LOD, limit of detection.
