# Supplementary material for: Facile Titrimetric Assay of Lysophosphatidic Acid in Human Serum and Plasma for Ovarian Cancer Detection
Source: J Cancer Prev. 2023 Jun 30;28(2):31–9. doi: 10.15430/JCP.2023.28.2.31 (PMC10331031; doi:10.15430/JCP.2023.28.2.31)
Supplement: Supplementary file 1 [file jcp-28-2-31-supple.pdf]

Titrimetric assay of unknown serum and plasma samples

Table S1 shows the concentration of lysophosphatidic acid (LPA) in unknown serum and plasma samples measured through titrimetric method.

Table S1. The concentration of lysophosphatidic acid in unknown serum samples of different ages groups of female

Age group (yr)	Sample					Mean value	± Standard deviation
	A	B	C	D	E		
21-25	1.87	1.37	2.18	4.37	0.93	2.144	1.332697
26-30	1.56	2.18	3.12	5	1.99	2.77	1.370766
31-35	1.87	4.37	2.5	1.25	0.78	2.154	1.397866
36-40	0.93	1.56	2.5	1.01	2.34	1.668	0.730253
41-60	0.8	2.5	1.09	1.87	3.75	2.002	1.18295

Optimization of LPA concentration in serum samples of volunteers of same age

Thirty samples were analysed, three of each age group and the concentration of LPA in serum samples were calculated.

Table S2. Comparison of lysophosphatidic acid (LPA) concentration in serum samples of same age of three volunteers

Age of female (yr)	Concentration of LPA in 1st volunteer (μM)	Concentration of LPA in 2nd volunteer (μM)	Concentration of LPA in 3rd volunteer (μM)	Mean value	± Standard deviation
21	0.93	1.09	1.56	1.193333	0.327465
22	1.56	1.87	1.3	1.576667	0.285365
23	1.09	1.56	1.87	1.506667	0.392726
24	1.6	0.93	1.3	1.276667	0.335609
25	0.78	1.09	0.93	0.933333	0.155027
26	0.62	0.78	0.54	0.646667	0.122202
27	3.12	2.18	0.5	1.933333	1.327303
28	3.75	0.18	1.3	1.743333	1.825824
29	1.09	1.99	1.01	1.363333	0.544181
30	1.3	1.56	1.09	1.316667	0.235443

Table S3. Concentration of lysophosphatidic acid in unknown plasma samples of female of different age groups

Age group in years	Sample					Mean value	± Standard deviation
	A	B	C	D	E		
21-25	1.25	2.81	2.05	3.31	2.05	2.294	0.791758
26-30	1.13	2.43	1.82	3.48	2.5	2.272	0.872451
31-35	1.52	1.92	2.25	1.69	1.25	1.726	0.381615
36-40	0.92	2.84	1.82	1.33	3.32	2.046	1.010584
41-60	1.99	2.34	2.69	3.4	1.03	2.29	0.876385

Comparison of LPA concentration in serum and plasma samples of same age female volunteers

The 10 samples of serum and plasma from healthy female volunteers of different age groups have been taken for analysis. The table below shows that the concentrations in the both samples are different.

Table S4. Comparison of lysophosphatidic acid (LPA) concentration in serum and plasma samples of same volunteers

S.No	Age of female (yr)	Concentration of LPA in serum (μM)	Concentration of LPA in plasma (μM)	Mean value	± Standard deviation
1	21	2.18	2.30	2.24	0.084853
2	22	2.38	2.53	2.455	0.106066
3	23	3.21	3.37	3.29	0.113137
4	24	1.87	2.05	1.96	0.127279
5	25	1.99	2.21	2.1	0.155563
6	26	1.32	1.44	1.38	0.084853
7	27	3.09	3.16	3.125	0.049497
8	28	1.87	1.92	1.895	0.035355
9	29	2.18	2.25	2.215	0.049497
10	30	2.76	2.84	2.8	0.056569
